# Distribution, Prevalence, and Antibiotic Susceptibility Profiles of Infectious Noncholera *Vibrio* Species in Malaysia

**DOI:** 10.1155/2023/2716789

**Published:** 2023-05-26

**Authors:** Murnihayati Hassan, Mohammad Ridhuan Mohd Ali, Hana Farizah Zamri, Nur Asyura Nor Amdan, Mohammad Noor Amal Azmai, Saraswathiy Maniam, Norfarrah Mohamed Alipiah, Rohaidah Hashim

**Affiliations:** ^1^Bacteriology Unit, Infectious Disease Research Center (IDRC), Institute for Medical Research (IMR), National Institutes of Health (NIH) Complex, Ministry of Health Malaysia, Setia Alam, 40170 Shah Alam, Kuala Lumpur, Selangor, Malaysia; ^2^Aquatic Animal Health & Therapeutics Laboratory (AquaHealth), Institute of Bioscience, Universiti Putra Malaysia, 43400 UPM Serdang, Selangor, Malaysia; ^3^Department of Biology, Faculty of Science, Universiti Putra Malaysia, 43400 UPM Serdang, Selangor, Malaysia

## Abstract

**Background:**

The noncholera *Vibrio* spp. which cause vibriosis are abundantly found in our water ecosystem. These bacteria could negatively affect both humans and animals. To date, there is a paucity of information available on the existence and pathogenicity of this particular noncholera *Vibrio* spp. in Malaysia in comparison to their counterpart, *Vibrio cholera*.

**Methods:**

In this study, we extracted retrospective data from Malaysian surveillance database. Analysis was carried out using WHONET software focusing noncholera *Vibrio* spp. including *Vibrio parahaemolyticus*, *Vibrio vulnificus*, *Vibrio fluvialis*, *Vibrio alginolyticus*, *Vibrio hollisae* (*Grimontia hollisae*), *Vibrio mimicus*, *Vibrio metschnikovii*, and *Vibrio furnissii*.

**Results:**

Here, we report the first distribution and prevalence of these species isolated in Malaysia together with the antibiotic sensitivity profile based on the species. We found that *V. parahaemolyticus* is the predominant species isolated in Malaysia. Noticeably, across the study period, *V. fluvialis* is becoming more prevalent, as compared to *V. parahaemolyticus*. In addition, this study also reports the first isolation of pathogenic *V. furnissii* from stool in Malaysia.

**Conclusion:**

These data represent an important step toward understanding the potential emergence of noncholera *Vibrio* spp. outbreaks.

## 1. Introduction

*Vibrio* spp. are ubiquitous in freshwater, estuarine, and saltwater aquatic milieus [1]. They are curved Gram negative bacilli, oxidase and catalase positive, and are generally sensitive to vibriostatic agents. Their motility is conferred by polar flagella [2]. *Vibrio* genus consists of about 150 species which can be further categorized into halophilic and nonhalophilic *Vibrio* spp. based on their sodium requirement. Although nonhalophilic *Vibrio* spp. in humans usually cause moderate infection, they are also known to cause outbreaks in humans, fish, and other aquatic animals with undesirable economical and public health implications [3, 4].

Human vibriosis can be classified into two major groups: cholera and noncholera. *Vibrio cholerae* is an important human pathogen responsible for cholera. Cholera is both waterborne and foodborne causing approximately 1.3 to 5 million cases reported annually with the death toll of 21,000–143,000 lives/year [5, 6]. *V. cholerae* serotypes O1 and O139 are exclusively accountable for cholera epidemics. Upon entry into the human host, *V. cholerae* adhere to intestinal epithelium followed by secretion and accumulation of cholera toxin (CT), which is responsible for intense watery diarrhea that potentially led to fatality. Virulence genes located in the ToxR regulon within *V. cholerae* genome are in particular responsible for severe symptoms of cholera [7]. Meanwhile, *V. cholerae* serotypes non-O1 and non-O139 are responsible for sporadic cholera-like infections, bacteremia, and septicemia [8–10]. The *V. cholerae* non-O1/non-O139 are categorized as noncholera *Vibrio cholerae*, as well as *V. cholerae* serotypes O1 and O139 that do not produce cholera toxin [11]. Such serotypes have been associated with clonal outbreak among diarrheal patients in Kolkata [12].

Other *Vibrio* spp., such as *V. mimicus*, *V. metschnikovii*, *V. vulnificus*, *V. alginolyticus*, *V. parahaemolyticus*, and *V. furnissii*, can also cause illnesses in humans known as vibriosis, which commonly is presented with gastrointestinal infection, wound infections, and septicemia [13]. Certain species such as *V. metschnikovii* have been isolated from a range of conditions such as septicemia and wound [14, 15]. Differently, cholera epidemics are highly associated with sanitary quality and socioeconomic status (typically transmitted from human to human or contaminated water). However, vibriosis caused by other *Vibrio* spp. is closely related to the consumption of contaminated shellfish or contaminated water and is independent of the sanitation status [6, 16, 17].

The presence and compositions of *Vibrio* spp. in aquatic habitats are dynamic and influenced by the geographical factor, climate, and salinity [18]. Therefore, analysis of local clinical and environmental data is necessary to develop an understanding of occurrences of vibriosis as a result of interaction between human health and other domains over time. This article aims to describe the current *Vibrio* spp. isolated from clinical samples in Malaysia from 2014 to 2020.

## 2. Materials and Methods

Nation-wide hospital antimicrobial susceptibility data between 2014 and 2020 were retrieved from Malaysia's local surveillance program. These data were catalogued in a downloadable database software developed by the World Health Organization (WHO), known as WHONET. The program converts various types of data from the different laboratory information systems of each hospital into a standardized common code and file format, enabling centralized data monitoring and analysis.

From 2014 to 2020, an average of 42 Malaysian hospitals participated in the WHONET-Malaysia network, contributing laboratory data consistently throughout the year. All antimicrobial tests and respective bacterial identification tests were performed in each hospital's clinical laboratories. This study focused on the available data associated with all noncholera *Vibrio* spp. reported within these seven surveillance years and their antimicrobial susceptibility profiles. In addition, *V. cholerae* nonO1/nonO139 was not included in this survey. The WHONET software provides basic statistical analysis of the input hospital data, with a special focus on the analysis of the antimicrobial susceptibility test results.

## 3. Results

Between 2017 and 2020, a total of 270 noncholera *Vibrio* spp. were isolated from 261 patients in Malaysia (Table 1). The distribution of samples was predominantly from stool (*n* = 137, 50.7%) and blood culture (*n* = 80, 29.6%). Among all, *V. parahaemolyticus* was the main species identified (*n* = 156, 57.8%), followed by *V. vulnificus* (*n* = 48, 17.8%), *V. fluvialis* (*n* = 30, 11.1%), *V. alginolyticus* (*n* = 16, 5.9%), and other *Vibrio* spp., namely, *V. hollisae (Grimontia hollisae)*, *V. mimicus*, *V. metschnikovii*, and *V. furnissii* (Table 1).

The antimicrobial susceptibility pattern from our data showed that *Vibrio* spp. isolated from clinical isolates during the period of the study remained susceptible to the common antibiotic regimes (Table 2). For instance, 91–100% of the isolates were sensitive to ceftazidime, gentamicin, imipenem, and meropenem. Similarly, for ciprofloxacin, 94–100% of the isolates were sensitive, except the *V. parahaemolyticus* (82%). On the other hand, in the absence of *ß*-lactamase inhibitor, the sensitivity of *V. parahaemolyticus* and *V. fluvialis* towards ampicillin was only 9% and 16%, respectively. In addition, high percentage of resistance was observed in *V. parahaemolyticus* against cefuroxime (68%) and *V. fluvialis* towards cefotaxime (50%), respectively.

**Figure 1:**
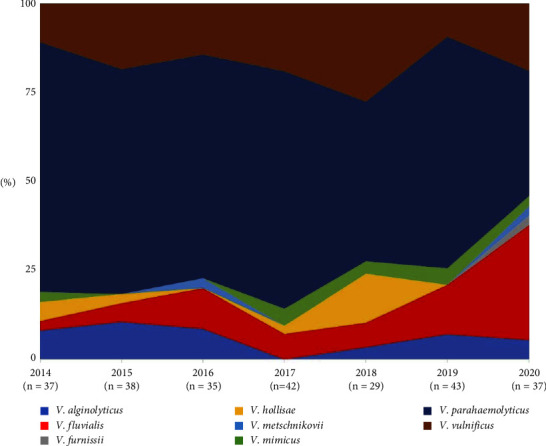
The pattern of noncholera *Vibrio* isolation between 2014 and 2020.

Across the 7-year surveillance (2014–2020), the average isolation of noncholera *Vibrio* was 37.3 cases/year (29–43 cases) (Figure 1). The data revealed that *V. parahaemolyticus* predominate the landscape of human infection during the period of the study. Surprisingly, *V. fluvialis was* increasingly isolated from clinical specimens from 3% in 2014 to 32% in 2020. Meanwhile, *V. furnissii*, *V. hollisae*, *V. metschnikovii*, and *V. mimicus* were not routinely isolated every consecutive year.

## 4. Discussion

*V. cholerae* is rarely isolated in Malaysia, yet it remains an important threat to human health in many developing countries [19]. Steadily, noncholera *Vibrio* spp. such as *V. fluvialis*, *V. mimicus*, *V. metschnikovii*, and *V. furnissii* become emerging causes of human infections often associated with foodborne diarrheal outbreaks. The clinical manifestations depend on the host susceptibility, route of infections, and inoculum size [20, 21]. In this study, it is shown that *V. parahaemolyticus* was the main causative agent of vibriosis in Malaysia, consistent with the other part of the world [22]. *V. parahaemolyticus* is an aquatic zoonotic pathogen that not only infects humans but is also a threat to aquaculture animal health. In aquaculture animals, *V. parahaemolyticus* causes early mortality syndrome (EMS) in shrimps and vibriosis in fishes with a high mortality rate and cause significant economic losses [23, 24]. Following the infection alarm in humans, isolates of *V. parahaemolyticus* from seafood in Selangor Malaysia have been found and characterized to be resistant to several antibiotics and possess at least one plasmid and positive *ToxR* genes [25].

In this study, *V. fluvialis* show an increasing trend during the study period. To date, there is scarce information available on human infections caused by *V. fluvialis* [26, 27]. *V. fluvialis* is similar to other noncholera *Vibrio* spp., they cause mild self-limiting gastrointestinal infections. Oral fluid replacement therapy is the mainstay of symptomatic treatment in mild cases. However, antimicrobial therapy using doxycycline or ciprofloxacin can be initiated in severe or prolonged cases [6]. On the other hand, *V. vulnificus* infection is associated with a higher mortality rate, especially in high-risk patients with treatment delay [28]. This study also had shown *V. vulnificus* as the second causative agent of vibriosis human infection in Malaysia. In the US, Brazil, Italy, and India, the species become a healthcare burden with a mortality rate more than 30% [29]. This organism is a zoonotic pathogen that can cause septicemia in fish, where iron in the blood signals the production of toxins that elevate the severity [30]. The acquirement of genes to evade host immune systems and antibiotic resistance are aggravated by uncontrolled usage of antibiotics in the aquaculture sectors [31].

Multidrug-resistant*Vibrio* spp. in the aquatic environment was common in fish farming [32, 33]. In Malaysia, it was reported that up to 80% of the *Vibrio* isolates from the west coast of Peninsular Malaysia were found to be resistant to one or more classes of antibiotics [34]. However, in this study, the vast majority of the clinical strains from human cases were sensitive to the cephalosporin and carbapenem class of antibiotics. Despite this observation, the retrieved surveillance data from human infections are limited by the number of isolates that were tested, hence may not represent the real clinical epidemiology. In some countries such as Bangladesh, China, and Vietnam, vibriosis is a significant public health problem, of which, outbreaks occur regularly, associated with wet season or summer with high dynamicity [35, 36]. For instance, *V. parahaemolyticus* serotype O10:K4, which have been frequently associated with outbreak in China, has spread to Thailand, with high sensitivities (89–100%) toward *ß*-lactam antibiotic such as ampicillin/sulbactam and ceftazidime [37, 38]. Overall, the incidence of noncholera vibriosis in Asia and Southeast Asia is likely to be underreported, as many cases are not diagnosed or notifiable to health authorities [6].

In summary, this study highlights *Vibrio parahaemolyticus* as a dominant *Vibrio* spp. causing vibriosis in Malaysia. Further analysis of the clinical significance of other *Vibrio* species that show emerging trends will require clinical data analysis and further laboratory characterization study.

## Figures and Tables

**Table 1:** Distribution of noncholera *Vibrio* spp. based on specimen types.

Organism	Type of specimen, *n* (%)														Total specimen, *n*(%)	Patien, t *n*(%)
	Bile	Blood	Ear	Eye	Fluid	Foot	Genital	Pus	Respiratory	Swab	Stool	Tissue	Urine	Unspecified		
*V. alginolyticus*		8	1				3				1	2	1		16 (5.9)	16 (6.1)
*V. fluvialis*	2	9			1				1	2	15				30 (11.1)	30 (11.5)
*V. furnissii*											1				1 (0.4)	1 (0.4)
*V. hollisae*		8													8 (3)	8 (3.1)
*V. metschnikovii*		1			1										2 (0.7)	2 (0.8)
*V. mimicus*		1		1				2			1	4			9 (3.3)	7 (2.7)
*V. parahaemolyticus*		18			2					3	118	2	4	9	156 (57.8)	154 (59.0)
*V. vulnificus*	1	35		1		1		1		1	1	2		5	48 (17.8)	43 (16.5)
Total	3 (1.1)	80 (29.6)	1 (0.4)	2 (0.7)	4 (1.5)	1 (0.4)	3 (1.1)	3 (1.1)	1 (0.4)	6 (2.2)	137 (50.7)	10 (3.7)	5 (1.9)	14 (5.2)	270	261

**Table 2:** Sensitivity of noncholera *Vibrio* spp. against selected antibiotics (% of sensitivity).

Organism/antibiotics	Ampicillin	Ampicillin/sulbactam	Amoxicillin/clavulanic acid	Cefuroxime	Ceftazidime	Cefotaxime	Cefepime	Imipenem	Meropenem	Amikacin	Gentamicin	Ciprofloxacin	Bactrim
*V. alginolyticus*	70		67	100	100	67	100	100	100	67	100	100	80
*V. fluvialis*	16	86	64	75	100	50	89	90	90	100	100	100	95
*V. furnissii*												100	100
*V. hollisae*			100	100	100	100	100	100	100	50	100		
*V. metschnikovii*					100				100	100		100	50
*V. mimicus*		100	33		100	100	100	100	100	100	100	100	78
*V. parahaemolyticus * ^1^	9	100	96	32	99	91	91	100	100	97	97	82	95
*V. vulnificus*	89	95	89	90	100	93	100	100	100	80	100	94	95

^1^Only this organism contains >50 isolates.

## Data Availability

The data used to support the findings of this study are available from the corresponding author on request.
